# Food craving, vitamin A, and menstrual disorders: A comprehensive study on university female students

**DOI:** 10.1371/journal.pone.0310995

**Published:** 2024-09-25

**Authors:** Liton Chandra Sen, Ishrat Jahan, Nadia Salekin, Jahid Hasan Shourove, Mosiur Rahman, Md Jamal Uddin, Cuilin Zhang, Davidson H. Hamer, G. M. Rabiul Islam

**Affiliations:** 1 Faculty of Nutrition and Food Science, Department of Community Health and Hygiene, Patuakhali Science and Technology University, Dumki, Patuakhali, Bangladesh; 2 Department of Food Engineering and Tea Technology, Shahjalal University of Science and Technology, Sylhet, Bangladesh; 3 Department of Population Science and Human Resource Development, University of Rajshahi, Rajshahi, Bangladesh; 4 Department of Statistics, Shahjalal University of Science and Technology, Sylhet, Bangladesh; 5 Faculty of Graduate Studies, Daffodil International University, Dhaka, Bangladesh; 6 Yong Loo Lin School of Medicine, National University of Singapore, Singapore, Singapore; 7 Department of Global Health, Boston University School of Public Health, Boston, MA, United States of America; 8 Department of Medicine, Section of Infectious Diseases, Boston University Chobanian & Avedisian School of Medicine, Boston, MA, United States of America; 9 Friedman School of Nutrition Science and Policy, Tufts University, Boston, MA, United States of America; Galgotias University, INDIA

## Abstract

**Background:**

Menstrual disorders, influenced by dietary habits like high fat intake and low fruit and vegetable consumption, are a global public health issue. This study assessed the prevalence of dysmenorrhea, premenstrual syndrome (PMS), and irregular menstrual cycle (IMC) among female university students in Bangladesh, focusing on food cravings and low vitamin A intake as risk factors.

**Methods:**

In this comprehensive study, data from randomly selected female university students were collected using a structured questionnaire. The associations were analyzed through chi-square tests and multivariable logistic regression, reported as adjusted odds ratios (AOR).

**Results:**

The most prevalent menstrual disorder was dysmenorrhea (68.3%) followed by PMS (33.8%), and IMC (24.3%). Food cravers for high-fat and sweet foods were likely to experience dysmenorrhea (AOR: 2.4, 95% CI: 1.5–3.9, P<0.001), suffer from PMS (AOR: 3.9, 95% CI: 2.3–6.6, P<0.001), and have IMC (AOR: 3.0, 95% CI: 1.6–5.3, P<0.001) vs. subjects who didn’t. Subjects consuming vitamin A-rich plant foods had 40% (AOR: 0.6, 95% CI: 0.4–0.9, *P* < 0.01) and 60% (AOR: 0.4, 95% CI: 0.2–0.6, *P*<0.001) less likely suffering from dysmenorrhea and IMC vs. who didn’t. Both underweight and overweight/obese subjects experienced more than 2-fold dysmenorrhea vs. normal-weight peers. The chance of IMC was nearly 3-fold among overweight/obese subjects. However, lower physical activity was associated with PMS and IMC whereas family history was associated with dysmenorrhea and PMS. Among the socio-demographic factors, maternal education, place of residence, and earlier menarche (≤12 years) were associated with dysmenorrhea while marital status was associated with IMC.

**Conclusion:**

This study indicates that increasing the intake of vitamin A-rich plant foods and reducing high-fat, sweet foods can lower the risk of dysmenorrhea and IMC. Additionally, it highlights the need for regular exercise to mitigate the increased risk of PMS and IMC.

## Introduction

Menstrual disorders are a common health problem among adolescents and young adult women affecting about 75% of adolescent girls and their quality of life [1]. While some women go through their normal monthly periods without complications, others experience some abnormalities that include but are not limited to dysmenorrhea, premenstrual syndrome (PMS), irregular menstrual cycle (IMC), and menorrhagia [2,3]. Dysmenorrhea, one of the most common menstrual disorders among adolescent girls and young adults, is characterized by menstrual cramps representing cyclic pelvic pain directly associated with menstruation. The pain severity is significantly associated with the excess production of prostaglandins (PG) in the endometrium during the ovulatory cycle [4]. PMS symptoms include mood swings, anxiety, irritability, tiredness or trouble sleeping, decreased interest in normal activities, concentration difficulties, bloating or tummy pain, craving for certain foods, and certain physical symptoms, e.g., joint or muscle pain, breast tenderness, headaches, spotty skin, swelling or bloating of the abdomen [5,6]. When menstruation does not take place at 28-day intervals, rather it occurs either in short or long time intervals, we define it as IMC. Heavy or prolonged menstrual bleeding is menorrhagia [7–9].

Numerous studies have demonstrated that various factors are associated with menstrual disorders, encompassing dietary diversity, food cravings, nutritional status, physical activity levels, caffeine consumption, cigarette smoking, obesity, depression, and socio-demographic factors [10–14]. Moreover, many earlier studies revealed that dysmenorrhea is associated with diet, body mass index (BMI), age (<30 years), age at menarche (<12 years), longer cycles, heavy menstrual flow, family history of dysmenorrhea and PMS, smoking, skipping breakfast, and socio-economic factors i.e., household income, mother’s education and place of residence, etc. [15–20]. The higher prevalence of PMS is associated with smoking and high-calorie content fat and sugar-based products as well as salty food consumption while physical exercise reduces the risk of having PMS [21,22]. Further, IMC is associated with obesity, smoking, psychological stress, anxiety, sleep problems, socio-demographic factors, low-quality diet, and low levels of physical activity [23–26]. Daily meat consumption increases the risk of IMC while fruits and vegetables reduce it [27]. In addition, genetic and environmental factors can influence the incidence of PMS. Unhealthy food habits such as highly processed fast foods can boost PMS risk [28]. In addition, consuming high dietary fat has been associated with PMS [29]. Some studies also reported that food containing refined sugar was associated with PMS [21,30,31]. Thus PMS is associated with the desire to eat junk food, meat, highly sweet foods, and sweet-food cravings [13,30]. Vitamin A, particularly in the form of carotenoids found in colorful fruits and vegetables, exhibits anti-inflammatory properties. Since dysmenorrhea involves uterine inflammation, consuming a diet high in anti-inflammatory compounds may potentially ease symptoms [32,33]. Research primarily emphasizes overall dietary patterns rather than singular nutrients like vitamin A in managing dysmenorrhea [34]. Nevertheless, plant-based diets abundant in vitamins and minerals have been shown to decrease inflammation and enhance general well-being, indirectly supporting menstrual health [35]. Besides, serum vitamin A is significantly associated with PMS and primary dysmenorrhea [32,36]. Though menstrual disorder is a common global problem among adolescents and young women, the risk factors emphasizing vitamin A-rich food consumption and appetite associated with all dimensions of menstrual disorder are not yet clear. In addition, there is an evidence gap in Bangladesh regarding the prevalence of menstrual disorders among young females and associated risk factors. Therefore, we aimed to delineate the prevalence of menstrual disorders and identify their associated risk factors, with a particular focus on the consumption of vitamin A-rich foods and appetite among university female students. We believe that this study enriches the understanding of menstrual abnormalities and may suggest strategies to combat the incidence of menstrual disorders during policy implementation.

## Methodology

### Data collection and sampling

**Fig 1:**
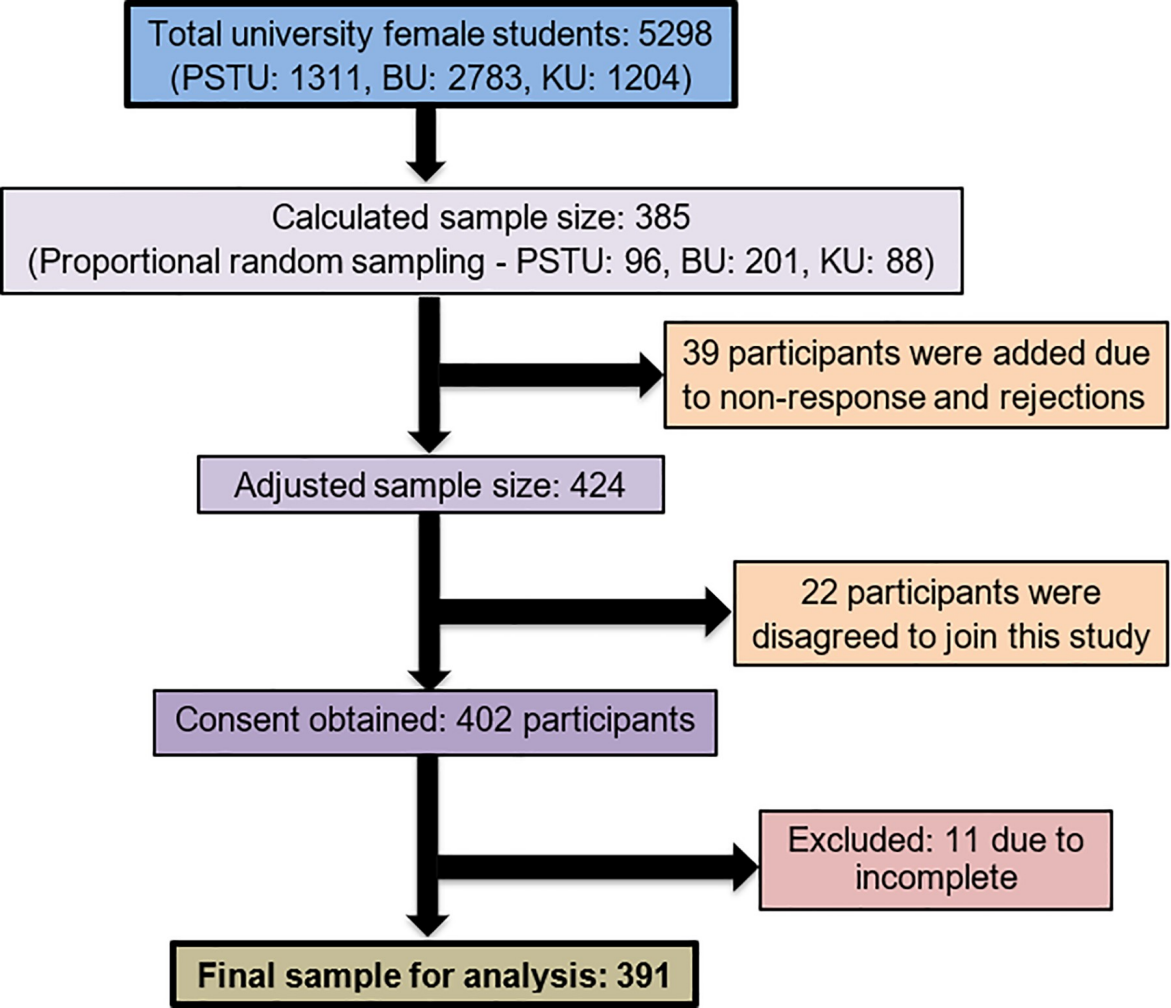
STROBE flow chart of the sample selection process.

This cross-sectional study was conducted among female students at randomly selected three universities in Bangladesh viz., Patuakhali Science and Technology University (PSTU), Barisal University (BU), and Khulna University (KU) from 15 November 2022 to 15 January 2023. During the survey period, a total of 1311, 2783, and 1204 female students were studying in PSTU, BU, and KU respectively. The sample size was calculated using the formula n = z^2^_*_p(1−p)/Ɛ; where we considered z for a 95% confidence level as 1.96, Ɛ as the margin of error of 5%, and p was assumed as the population proportion 0.5. Thus, the sample size came out to be 385. Using proportional random sampling, 96 students from PSTU, 201 from BU, and 88 from KU were selected. To account for potential non-respondents and rejections (approximately 10%), the sample size was increased to 424 participants. Prior to administering the survey, we secured approval from the university’s higher authorities. We developed a questionnaire according to Nooh et al. and modified based on the Bangladesh context [37]. The questionnaire was developed in English and then translated into Bangla, the national language of Bangladesh due to the absence of international students. A reverse translation was also made to confirm the originality **(S1 File)**. Two female interviewers were assigned and trained to assist in data collection as the participants felt comfortable with the female interviewer giving information regarding their menstruation. A pilot study was performed among 50 students to reflect our main study as well as to validate the questionnaire and assess the study’s feasibility. After explaining the study’s purpose and obtaining consent, 402 participants agreed to join. Subsequently, 391 respondents were included in the analysis, as the responses of 11 subjects were excluded due to incomplete or inappropriate responses. The entire sampling method is presented in **Fig 1.**

### Outcome variables

In this study, dysmenorrhea, PMS, and IMC were used as outcome variables. The severity of dysmenorrhea was measured through a multidimensional scoring system and categorized into four grades none, mild, moderate, or severe pain **(S1 Table)**. This grading was based on the intensity of the pain, its impact on daily activities, and the requirement for analgesics [38]. In the case of PMS, the participants were questioned about whether they had experienced depression, rapid mood changes, anxiety, irritability, change in appetite, painful or tender breasts, and swelling or bloating of the abdomen around 7–10 days prior to the start of their period with cessation of these symptoms during their period [37,39]. Additionally, the IMC was defined as a varying cycle length of less than 21 days or more than 35 days [7,8].

### Explanatory variables

As a key explanatory variable, we assessed whether respondents experienced food cravings, defined as an intense desire to consume specific foods, including snacks, chocolate, sweets, and other foods, or any changes [13]. Dietary diversity along with vitamin A consumption from plant and animal food sources were computed based on the guidelines of the Food and Agriculture Organization (FAO) **(S2 File)** [40].

In addition, some socio-demographic and nutritional information were used as related explanatory variables viz., father’s and mother’s educational level, place of residence, father’s and mother’s occupation categorized as formal occupation that includes government job, private job, and retired persons while informal occupation encompasses entrepreneur, fisherman, farmers, homemaker; marital status and age at menarche categorized as ≤12 years, >12 years [36]. The BMI (kg/m^2^) was used as a nutritional index to measure obesity. The BMI categories used cut-off points recommended for the Asian population proposed by the World Health Organization viz., i) underweight (<18.5) ii) normal-weight (18.5–22.9), iii) overweight (23.0–27.5), and iv) obese (>27·5) [41,42]. Because only 2.55% (n = 10) of the studied respondents were classified as obese, for the regression model in this study, the overweight/obese groups were combined to create an overweight/obese category to avoid problems associated with zero cell counts in estimating the models. Nonetheless, the physical activity of the respondents was categorized as i) sedentary (typical activities of daily living with little or no exercise), ii) active (person running one hour daily) and athlete (running, fast cycling, jumping, climbing, swimming, sports, etc.). Moreover, we also included whether the participants were infected by COVID-19 and had a family history of menstrual disorders as explanatory variables.

### Ethical consideration

The study was conducted in accordance with the ethical standards of the School Ethics Review Committee (SERC) on human experimentation and with the Declaration of Helsinki (1964), as revised in 2000 [43]. Ethical approval for the study protocol, questionnaire, and written consent form was obtained from the Shahjalal University of Science and Technology (SUST) Research Ethics Board (ref.no. AST/002/258, October 24, 2022). Before the interview began, participants were informed about the study’s objectives and their right to withdraw at any time. They were also asked for permission to use the collected data for publication, reproduction, broadcast, and other purposes. Participants were assured that all data would remain strictly confidential and used only for research purposes. Those who consented signed a written consent form. Each interview lasted 20 minutes, during which the interviewers filled out and promptly collected the questionnaires to avoid contamination and maintain confidentiality. To ensure anonymity, all personal information was anonymized.

### Statistical analysis

Pearson’s Chi-square (**χ**^2^) test was performed to determine statistically significant differences observed within the categories of outcome variables in relation to explanatory variables. The likelihood-ratio test was employed when more than 25% of the total cells had an expected frequency of less than 5. The ordinal logistic regression model was used to identify the risk factors associated with varying severity levels of dysmenorrheal pain. Logistic regression was employed to identify potential risk factors for PMS and IMC. The explanatory variables that had a significant association with dysmenorrhea pain, PMS, and IMC, determined through the **χ**^2^ or likelihood ratio test (**Table 1**), were included in the multivariate regression models in **Figs 3–5,** respectively. We also performed bivariate analysis **(S2–S4 Tables)**. The adjusted odds ratio (AOR) and 95% CI were used where appropriate and *P* < 0.05 was considered to show a statistical significance. The data were analyzed using the Stata software, version 17.0.

**Table 1:** Female university students with menstrual disorders stratified by explanatory variables.

Variables	Overall (N = 391)	Severity of dysmenorrhea pain					Premenstrual syndrome (PMS)			Irregular menstrual cycle		
		No Pain	Mild Pain	Moderate Pain	Severe Pain	χ^2^	Absent	Present	χ^2^	No	Yes	χ^2^
		n = 124	n = 183	n = 50	n = 34		n = 259	n = 132		n = 296	n = 95	
**Food habit**												
***Food craving (high fat and sweet food)***												
No	303 (77.4)	110(28.1)	146 (37.3)	33 (8.4)	14 (3.6)	39.0***	225 (57.5)	78 (19.9)	38.7***	250 (63.9)	53 (13.5)	33.9***
Yes	88 (22.6)	14 (3.6)	37 (9.5)	17 (4.4)	20 (5.1)		34 (8.7)	54 (13.9)		46 (11.8)	42 (10.8)	
***Dietary diversity score***												
Low (≤3 food groups)	46 (11.8)	13 (3.3)	24 (6.1)	5 (1.3)	4 (1.02)	2.3	36 (9.2)	10 (2.6)	3.4	38 (9.7)	8 (2.05)	2.5
Medium (4–5 food groups)	230 (58.8)	78 (19.9)	105 (26.9)	27 (6.9)	20 (5.1)		149 (38.1)	81 (20.7)		176 (45.01)	54 (13.6)	
High (≥6 food groups)	115 (29.4)	33 (8.4)	54 (13.8)	18 (4.6)	10 (2.6)		74 (18.9)	41 (10.5)		82 (21.0)	33 (8.4)	
***Consume vitamin A-rich plant food sources***												
No	181 (46.3)	33 (8.4)	101 (25.8)	21 (5.4)	26 (6.7)	38.0***	112 (28.6)	69 (17.7)	2.9	114 (29.2)	67 (17.1)	29.6***
Yes	210 (53.7)	91 (23.3)	82 (21.00)	29 (7.4)	8 (2.05)		147 (37.6)	63 (16.1)		182 (46.5)	28 (7.2)	
***Consume vitamin A-rich animal food sources***												
No	126 (32.2)	36 (9.2)	65 (16.6)	17 (4.4)	8 (2.05)	2.7	81 (20.7)	45 (11.5)	0.3	107 (27.4)	19 (4.8)	8.6**
Yes	265 (67.8)	88 (22.5)	118 (30.2)	33 (8.4)	26 (6.7)		178 (45.5)	87 (22.3)		189 (48.3)	76 (19.5)	
**Socio-demographic factors**												
***Father’s educational status***												
Below secondary (0–5 y schooling)	20 (5.1)	7 (1.8)	12 (3.07)	1 (0.2)	0 (0.0)	5.7^a^	14 (3.6)	6 (1.5)	0.1	15 (3.8)	5 (1.3)	0.005^a^
Secondary/Higher (>5 y schooling)	371 (94.9)	117(29.9)	171 (43.8)	49 (12.5)	34 (8.7)		245 (62.7)	126 (32.2)		281 (71.9)	90 (23.02)	
***Mother’s educational status***												
Below secondary	187 (47.8)	26 (6.7)	110 (28.1)	27 (6.9)	24 (6.1)	54.7***	116 (29.7)	71 (18.1)	2.8	144 (36.8)	43 (11.0)	0.3
Secondary/Higher	204 (52.2)	98 (25.06)	73 (18.7)	23 (5.9)	10 (2.6)		143 (36.6)	61 (15.6)		152 (38.9)	52 (13.3)	
***Place of residence***												
With family	47 (12.0)	30 (7.7)	12 (3.07)	4 (1.02)	1 (0.2)	26.0***	33 (8.4)	14 (3.6)	0.4	33 (8.4)	14 (3.6)	0.9
At student dormitory	344 (88.0)	94 (24.04)	171 (43.7)	46 (11.8)	33 (8.4)		226 (57.8)	118 (30.2)		263 (67.3)	81 (20.7)	
***Father’s occupation***												
Formal occupation	259 (66.2)	85 (21.7)	124 (31.7)	29 (7.4)	21 (5.4)	2.3	170 (43.5)	89 (22.7)	0.1	198 (50.6)	61 (15.6)	0.2
Informal occupation	132 (33.8)	39 (10.00)	59 (15.09)	21(5.4)	13 (3.3)		89 (22.8)	43 (11.0)		98 (25.06)	34 (8.7)	
***Mother’s occupation***												
Formal occupation	68 (17.4)	21 (5.4)	33 (8.4)	8 (2.05)	6 (1.5)	0.1	49 (12.5)	19 (4.9)	1.25	46 (11.8)	22 (5.6)	2.9
Informal occupation	323 (82.6)	103 (26.3)	150 (38.3)	42 (10.7)	28 (7.2)		210 (53.7)	113 (28.9)		250 (64.0)	73 (18.6)	
***Marital status***												
Ever married	33 (8.4)	10 (2.6)	18 (4.6)	3 (0.7)	2 (0.5)	1.20^a^	24 (6.1)	9 (2.3)	0.67	29 (7.4)	4 (1.02)	4.2*
Never Married	358 (91.6)	114 (29.1)	165 (42.2)	47 (12.02)	32 (8.2)		235 (60.1)	123 (31.5)		267 (68.3)	91 (23.3)	
***Age at menarche (years)***												
≤ 12 years	240 (61.4)	64 (16.4)	113 (28.9)	35 (9.0)	28 (7.1)	12.9**	148 (37.9)	92 (23.5)	5.8*	175 (44.8)	65 (16.6)	2.6
> 12 years	151 (38.6)	60 (15.4)	70 (17.9)	15 (3.8)	6 (1.5)		111 (28.4)	40 (10.2)		121 (31.00)	30 (7.6)	
***BMI (kg/m*** ^***2***^ ***)***												
Underweight (<18.5)	64 (16.4)	14 (3.6)	29 (7.4)	11 (2.8)	10 (2.6)	50.3***	44 (11.3)	20 (5.1)	2.8	47 (12.02)	17 (4.4)	31.06***
Normal weight (18.5–22.9)	212 (54.2)	97 (24.8)	78 (20.0)	23 (5.9)	14(3.5)		146 (37.3)	66 (16.9)		182 (46.6)	30 (7.6)	
Overweight/obese (>22.9)	115 (29.4)	13 (3.3)	76 (19.4)	16 (4.09)	10 (2.6)		69 (17.6)	46 (11.8)		67 (17.1)	48 (12.3)	
***Physical activity***												
Sedentary	310 (79.3)	99 (25.3)	147 (37.6)	36 (9.2)	28 (7.2)	2.00	193 (49.4)	117 (29.9)	10.6**	222 (56.8)	88 (22.5)	13.6***
Active and Athlete	81 (20.7)	25 (6.4)	36 (9.2)	14 (3.6)	6 (1.5)		66 (16.9)	15 (3.8)		74 (18.9)	7 (1.8)	
***COVID-19 Infection***												
No	301 (77.0)	105 (26.9)	141(36.1)	30 (7.6)	25 (6.4)	12.5**	212 (54.2)	89 (22.8)	10.3**	228 (58.3)	73 (18.7)	0.001
Yes	90 (23.0)	19 (4.9)	42 (10.7)	20 (5.1)	9 (2.3)		47 (12.0)	43 (11.0)		68 (17.4)	22 (5.6)	
***Family history of menstrual disorders***												
No	308 (78.8)	114 (29.2)	140 (35.8)	31 (7.9)	23 (5.9)	24.3***	230 (58.8)	78 (20.0)	46.2***	238 (60.9)	70 (19.9)	1.9
Yes	83 (21.2)	10 (2.6)	43 (11.00)	19 (4.8)	11 (2.8)		29 (7.4)	54 (13.8)		58(14.8)	25 (6.4)	

* Indicated chi-square values define the level of significance i.*e*. * p<0.*05*, ** p<0.*01*, *** p <0.*001 and data were presented as n (%)*. ^a^ *The likelihood-ratio chi2 value was employed as more than 25% of the total cells had an expected frequency of less than 5*.

## Results

### Descriptive analysis

**Fig 2:**
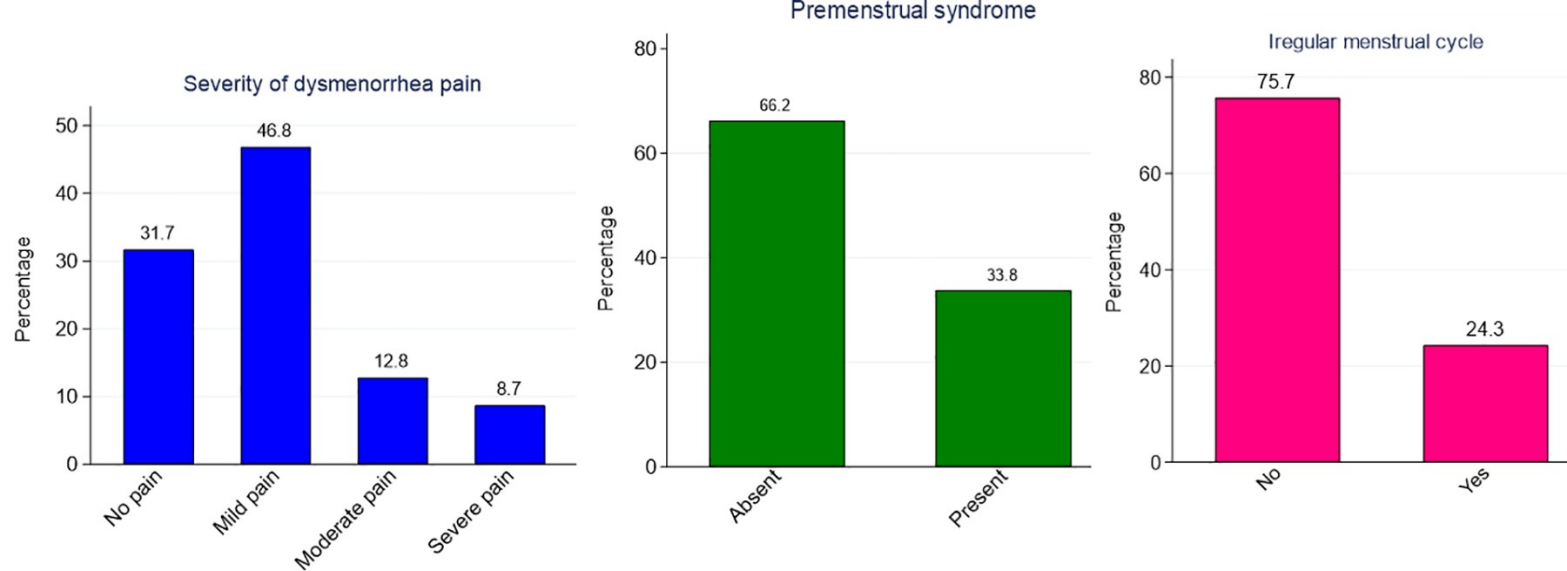
Prevalence of menstrual disorders (dysmenorrhea, premenstrual syndrome and irregular menstrual cycle since menarche) among female university students in Bangladesh.

The prevalence of menstrual disorders among university female students is also presented in **Fig 2**. In this study, 68.3% of the respondents reported having dysmenorrhea out of which 46.8% experienced mild, 12.8% with moderate, and 8.7% with severe pain. It was also evident that 33.8% of the respondents experienced one or more symptoms of PMS whereas IMC symptoms were present in 24.3% of the subjects.

**Table 1** presents key insights gathered from the survey data. It reveals that 22.6% of respondents experienced cravings for high-fat and sweet foods. Regarding dietary habits, 53.7% consumed vitamin A-rich plant foods, while a higher percentage, 67.8%, opted for vitamin A-rich animal-based foods within 24 hours of interview time. Regarding living arrangements, 12.0% of subjects resided with their families, while the majority of female respondents lived in student dormitories. Early menarche (≤ 12 years) was prevalent among 61.4% of the surveyed individuals. The descriptive analysis also noted that 16.4% of respondents were underweight, whereas 29.4% were classified as overweight or obese. In terms of physical activity, 79.3% led sedentary lifestyles, contrasting with 20.7% who maintained an active or athletic lifestyle. Moreover, 23.0% reported having contracted COVID-19, while 21.2% had a family history of menstrual disorders.

**Table 1** summarizes the results of the **χ**2 analysis. In this study, the **χ**2 analysis revealed significant associations between food cravings and all outcome variables (*P*<0.001). Consumption of vitamin A-rich plant food sources was found to be associated with dysmenorrhea (*P*<0.001) and IMC (*P*<0.001), whereas vitamin A consumption from animal food sources was significantly related to IMC (*P*<0.01) only. The results further indicated that the mother’s educational status and place of residence were significantly associated with dysmenorrhea (*P*<0.001), while marital status among students was linked to IMC (*P*<0.05). Age at menarche was significantly associated with dysmenorrhea (*P*<0.01) and PMS (*P*<0.05). BMI was significantly linked to dysmenorrhea (P<0.001) and IMC (P<0.001), while physical activity demonstrated a significant relationship with the occurrence of PMS (P<0.01) and IMC (P< 0.00). Additionally, COVID-19 infection status and family history of menstrual disorders showed strong associations with dysmenorrhea (*P*<0.01 and *P*<0.001 respectively) and PMS (*P*<0.01 and *P*<0.001 respectively).

### Dysmenorrhea pain and associated risk factors

**Fig 3:**
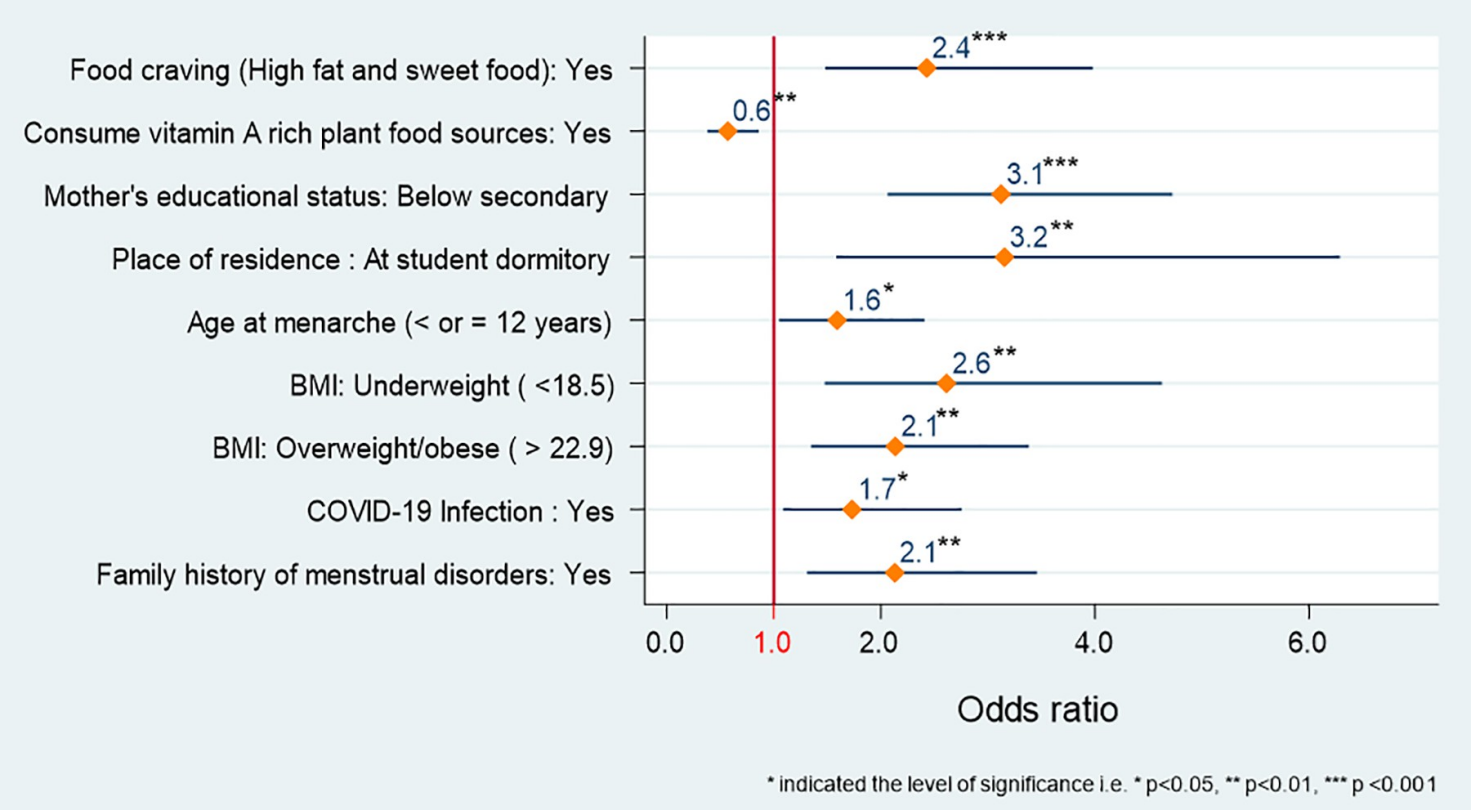
Association between explanatory variables and dysmenorrhea pain (the probability of being mild, moderate and severe dysmenorrhea pain vs no dysmenorrhea pain in the relevant category of explanatory variables).

The result of the association between dysmenorrhea pain and associated risk factors obtained through the ordinal logistic regression model is presented in **Fig 3**. The results illustrate that food craving was associated with increased dysmenorrheal pain while consuming vitamin A-rich plant food significantly reduces the dysmenorrhea pain. The girls who had food cravings for high-fat and sweet food were more prone to mild, moderate, and severe dysmenorrheal pain compared to those who had no food cravings (AOR: 2.4, 95% CI: 1.48–4.0, *P* < 0.001). Subjects who consumed vitamin A-rich plant foods were 40% less likely to be affected by dysmenorrheal pain (AOR: 0.6, 95% CI: 0.4–0.9, *P* < 0.01).

The analysis also demonstrated that there was a strong association between some socio-demographic factors (mother’s educational status, place of residence, and age at menarche) and dysmenorrhea. The respondents whose mothers had below secondary education had a 3-fold higher risk of suffering from mild, moderate, and severe dysmenorrheal pain compared to those whose mothers had secondary/higher education (AOR: 3.1, 95% CI: 2.06–4.7, *P* <0.001). The subjects who were away from home and lived at the student dormitory were 3 times more likely to suffer from dysmenorrhea than those who lived with their family (AOR: 3.1, 95% CI: 1.6–6.2, *P* < 0.01). The age at menarche significantly influenced the likelihood of experiencing dysmenorrheal pain. The respondents whose menstruation commenced at or before age 12 years were 60% more likely affected by dysmenorrheal pain vs. those above 12 years (AOR: 1.60, 95% CI: 1.05–2.4, *P* < 0.05).

There was also a strong relationship between BMI and the severity of dysmenorrheal pain. Subjects categorized as underweight and overweight/obese were 2.6 (AOR: 2.6, 95% CI: 1.5–4.6, *P* <0.01) and 2.1 (AOR: 2.1, 95% CI: 1.6–3.4, *P* <0.01) times more likely to have mild, moderate and severe dysmenorrheal pain compared to those having normal weight, respectively. Moreover, the subjects with COVID-19 infection and having a family history of menstrual disorders had 70% (AOR: 1.7, 95% CI: 1.09–2.8, *P* <0.05) and 2 times (AOR: 2.1, 95% CI: 1.3–3.5, *P* <0.01) higher chance of having mild, moderate and severe dysmenorrheal pain respectively.

### PMS and associated risk factors

**Fig 4:**
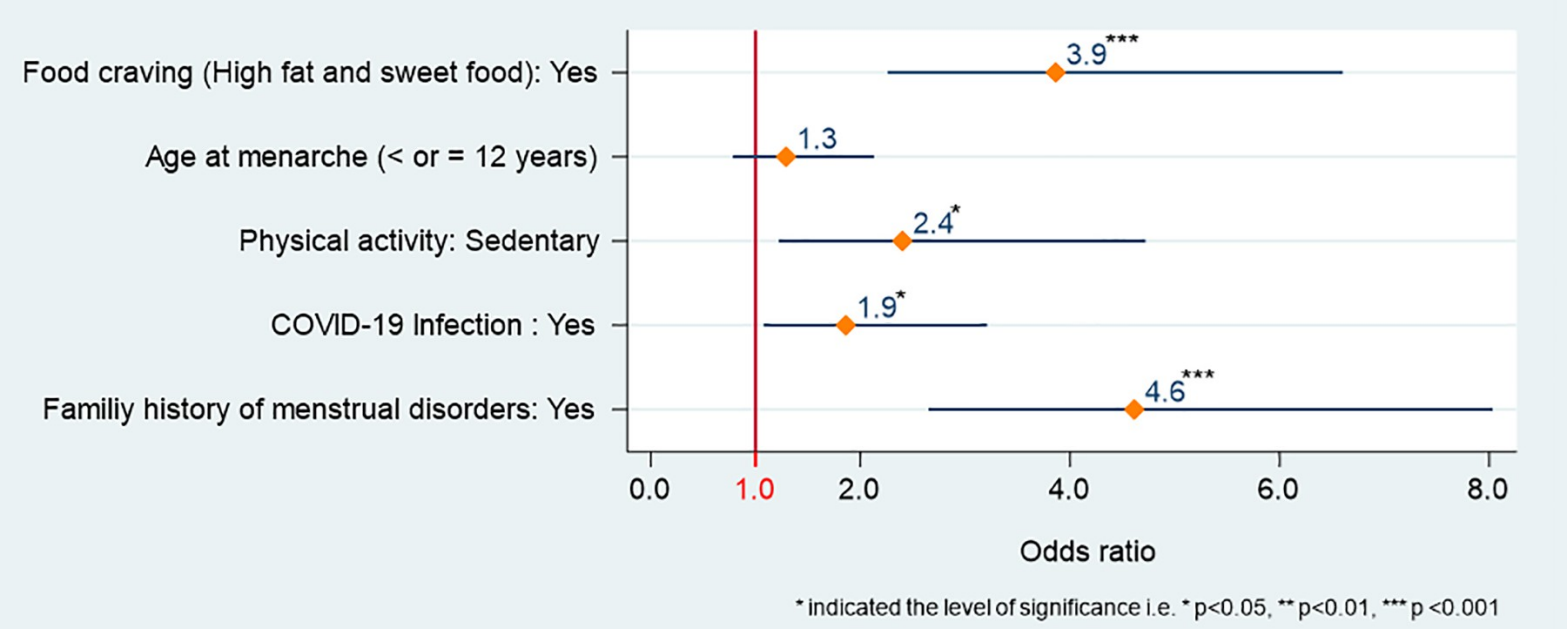
Association between explanatory variables and premenstrual syndrome.

**Fig 4** represents the results of the association between PMS and potential risk factors obtained through the binary logistic regression model. Like dysmenorrhea, PMS also exhibited a strong association with food cravings.

Among respondents who had high food cravings, approximately 4 times more experienced with PMS compared to those who have no food craving (AOR: 3.9, 95% CI: 2.3–6.6, *P* <0.001). Physical activity emerged also as a risk factor for triggering PMS. The respondents who were sedentary versus those belonging to active and athletic groups were 2 times more likely to experience PMS (AOR: 2.4, 95% CI: 1.2–4.7, *P* <0.05). Nevertheless, the results also illustrated that the subjects infected by COVID-19 had 2-times more chance of having PMS (AOR: 1.9, 95% CI: 1.08–3.2, *P* <0.05). Similarly, the odds of being exposed to PMS were 4 times higher in subjects having a family history of menstrual disorders (AOR: 4.6, 95% CI: 2.7–8.03, *P* <0.001).

### IMC and associated risk factors

**Fig 5:**
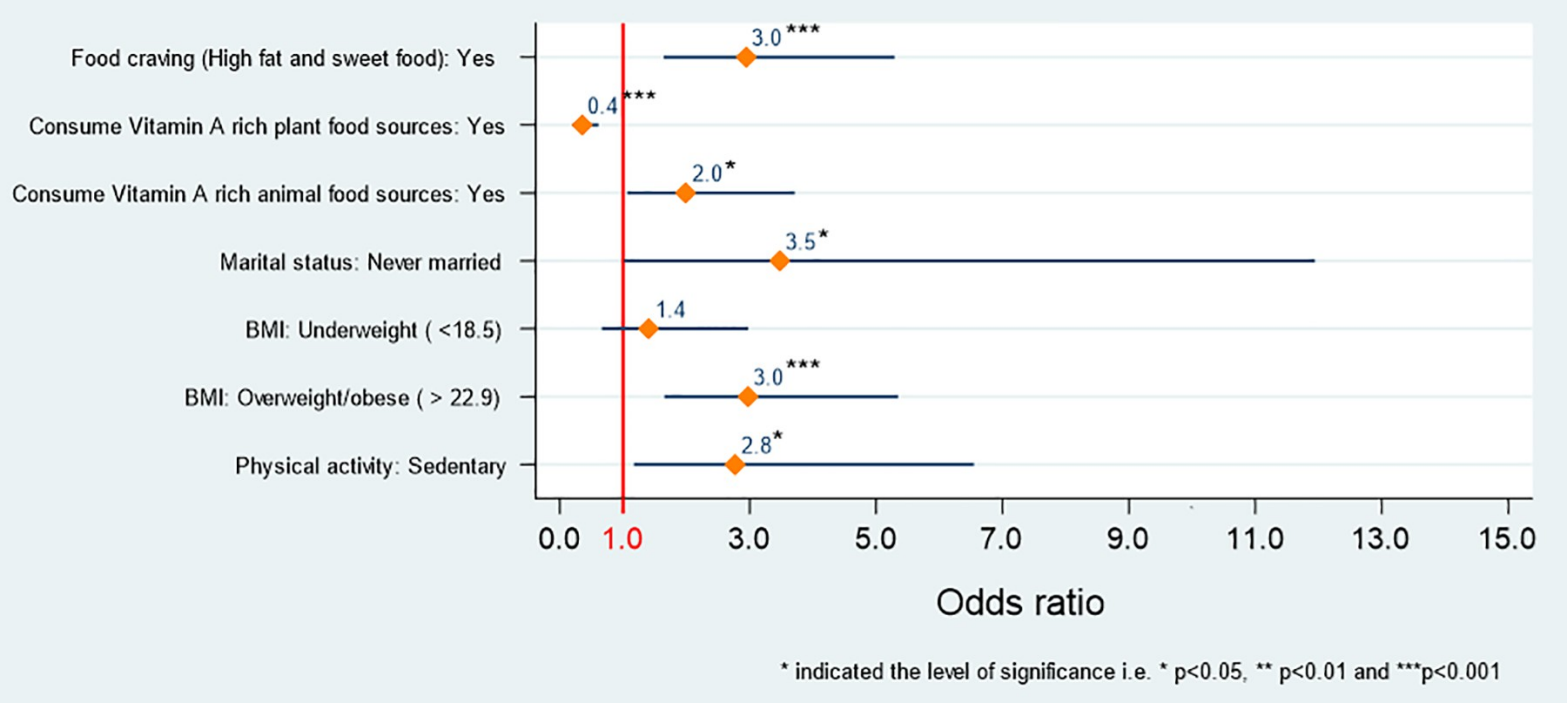
The association between explanatory variables and irregular menstrual cycle (IMC).

The multivariate results of IMC and associated risk factors are presented in **Fig 5**. Like dysmenorrhea and PMS, food cravings also played a significant role in mounting the occurrence of IMC. The likelihood of experiencing IMC was three times higher (AOR: 3.0, 95% CI: 1.6–5.3, *P* < 0.001) among food cravers compared to those without cravings. The subjects who consumed vitamin A-rich plant foods exhibited 60% less likely to suffer from IMC than those who didn’t (AOR: 0.4, 95% CI: 0.2–0.6, *P* < 0.001). Conversely, respondents who consumed vitamin A-rich animal-based foods were twice as likely to have IMC compared to those who did not consume these foods. (AOR: 2.0, 95% CI: 1.07–3.7, *P* < 0.05). The never married subjects had a 3 times higher risk of IMC compared to ever married subjects (AOR: 3.5, 95% CI: 1.02–12.0, *P* < 0.05). Overweight/obese respondents had three times the risk of IMC compared to those of normal weight (AOR: 3.0, 95% CI: 1.7–5.4, *P* < 0.001). Subjects who were sedentary in terms of physical activity had a significantly higher likelihood of developing IMC compared to those who were active and athletic (AOR: 2.8, 95% CI: 1.2–6.6, *P* <0.05).

## Discussion

This study examines the risk factors contributing to menstrual disorders among female university students. Notably, it represents the first study of its kind in Bangladesh. Previous research in the region has primarily focused on the prevalence of menstrual disorders and menstrual hygiene management among adolescent girls, overlooking the investigation of underlying risk factors associated with these disorders. This study provides evidence that the prevalence of menstrual disorders is high in Bangladesh. Moreover, it reveals insights into dietary habits such as food cravings and vitamin A consumption among the study subjects, highlighting potential associations with menstrual disorders [44–46].

A large portion of the respondents were suffering from dysmenorrhea (68.3%). This result aligns with previous records, which reported the prevalence of dysmenorrhea ranging from 60.9% to 72.0% in various countries around the world [37,46–48]. This study also noted that 33.8% of participants had PMS, a proportion that coincided with the findings of Hashim *et al*. who reported 35% of UAE university going students have experienced PMS [21]. In contrast, the prevalence of PMS ranged between 40.4% and 93.8% in several other countries [47–50]. Earlier investigations found that the prevalence of IMC varied between 8.4% and 24.4% globally [7,37,51,52] which was in with our study result. Exceptionally, studies from Italy and Lebanon, revealed that the incidence rate of IMC among adolescent and young women was as high as 72.8% and 59.4% respectively [3,50].

This study illustrates that food craving plays an important role in all forms of menstrual disorders. This relationship depicts that the food cravers are more prone to experience dysmenorrhea, PMS, and IMC, findings which are consistent with the results of other investigations [13,16,21,31,53]. Food cravings, which occur more frequently in women, often arise in response to emotional states, especially negative moods such as stress, anxiety, or sadness [54]. These cravings are not only driven by biological signals of hunger and satiety but also influenced by the desire for pleasurable or comforting sensations associated with certain foods, like taste and appearance [55]. This suggests that, for many women, food acts as a coping mechanism to temporarily alleviate negative emotions. Consequently, there is a strong association between mood swings and food cravings, emphasizing the psychological aspects of eating behavior in response to emotional states [56]. In fact, high-fat and sweet food consumption is associated with irritability, depression, anger, and sleeping disorders, all of which may act as a foundation of PMS [13]. The higher release of progesterone and lower level of estrogen during the late luteal phase contribute to a desire to consume more high-fat and sweet foods [31,57,58]. Moreover, high-fat and sweet foods tend to encourage hyperphagia, leading to an abnormally increased appetite [59]. The high-fat and sweet foods have a significant association with reward sensitivity, positive implicit attitude, and emotional and craving response during the luteal phase of the menstrual cycle especially among the women with PMS [30,31]. Indeed, during the late luteal phase, emotional response and food cravings usually increase and thereby decrease the quality of life and leading to a higher incidence of dysmenorrhea, PMS and irregular menstruation [17,21,28].

Our study elucidates that the consumption of vitamin A-rich plant foods reduces the risk of having mild, moderate, and severe dysmenorrheal pain and the probability of having IMC while participants who consume vitamin A-rich animal foods have a higher risk of IMC. Many previous studies show that consuming plant-based foods plays a protective role against dysmenorrheal pain [12,16,33,60,61]. Consuming eggs, fish, milk, and dairy products also reduce the dysmenorrheal pain [33,60]. However, our study shows that the consumption of vitamin A-rich animal foods increases the risk of IMC which support the finding of Wang et al. [62]. Some researchers suggest that vitamin A may have anti-inflammatory properties, which may potentially help alleviate symptoms of dysmenorrhea [63]. However, the evidence regarding the role of vitamin A consumption from plant and animal food sources influencing menstrual disorders among young women is still limited in literature and demands further research. Bahrami et al. reported that high serum vitamin A levels significantly reduced dysmenorrheal pain and PMS among adolescent girls in Iran while there was no significant association with IMC [36]. In a review, Fjerbaek and Knudsen reported that a high intake of ham, beef, and other red meat increased the risk of dysmenorrhea [61]. Red meat consumption is not directly linked to dysmenorrhea. However, certain components found in red meat, like saturated fats and oils, can contribute to inflammation, which might exacerbate the dysmenorrheal pain [16]. Additionally, red meat contains arachidonic acid, which can lead to increased production of prostaglandins, hormone-like substances that play a role in triggering uterine contractions and thus may contribute to cramping during menstruation [64,65]. However, the relationship between diet and menstrual pain can vary greatly among individuals.

Our study reveals that higher consumption of vitamin A from plant-based food reduces the risk of IMC. Usually, vitamin A is involved in the regulation of reproductive hormones by acting on the hypothalamo-pituitary-adrenal (HPA) axis and therefore deficiencies in vitamin A may be associated with menstrual irregularities [66]. Consumption of the Mediterranean diet, i.e., low consumption of animal source foods such as red and processed meats, white meat, and fish, and high consumption of fruits and vegetables decreases the risk of IMC [12]. Earlier studies reported that animal food sources played as a risk factor in causing IMC [62,67,68]. Our study illustrated that consumption of vitamin A-rich animal food sources had higher odds of having IMC. This may be because, heavy meat intake can disrupt the production of gonadotropin hormones, which are essential for regulating the menstrual cycle. This disruption can impair the follicular development, a crucial process for ovulation. As a result, menstrual cycles may become longer and more irregular [27,69].

Maternal education level appears to play an important role in dysmenorrhea. Habibi et al. and Adib-Rad et al. reported that the increased years of mother’s education was significantly associated with decreased severity of dysmenorrhea pain; their results are consistent with our findings [20,70]. Educated women are more likely to participate in decision-making process and choose healthier lifestyles, e.g., better dietary habits, physical exercise, assessing health care facilities, and menstrual hygiene practices which influence their daughters to maintain good menstrual health. In this study, place of residence appears as a significant factor associated with dysmenorrhea. The subjects living in dormitories were more vulnerable to dysmenorrhea, a result in line with the findings of Karout et al. in Lebanon [50], though Habib et al., found a reverse association in Iran [20]. However, the students who lived with their families were more likely to have healthier lifestyles compared to those living in dormitories like gypsy people. At home, they have access to healthy foods, regular sleep schedules, and opportunities for mental relaxation, all of which are scarce in dormitory life in Bangladesh.

Our study also reveals that unmarried subjects suffered more from IMC than others, a finding that is similar to a previous study [50]. Unmarried women suffering from psychiatric stress are a well-known factor contributing to IMC [26]. Besides, after marriage, the women pass through certain changes in the reproductive endocrine system which may influence the regular menstruation of married women [71]. The subjects with earlier menarche (≤12 years) had a higher risk of suffering from dysmenorrhea is supported by several studies [18,24,38,72,73]. Early onset of menstruation, accompanied by hormonal changes, can intensify both the severity and frequency of menstrual pain. Consequently, girls experiencing early maturation are at higher risk of developing menstrual disorders, such as the common occurrence of dysmenorrhea [51].

In this study, a U-shaped association was found between BMI and dysmenorrhea meaning that both underweight and overweight/obese subjects had higher odds of experiencing dysmenorrhea compared to those with normal weight which aligns with findings from other studies [74,75]. However, other studies have reported a higher risk of dysmenorrhea only among underweight subjects [15,18,19,76]. In contrast, Harlow and Park showed that overweight women have significant experience with dysmenorrheal pain [77]. However, the underlying pathophysiological mechanisms of this association are still unclear and they may differ in underweight and overweight females. After completion of growth, a critical weight (26–28% body fat) in women is very important for the onset and maintenance of the regular ovulatory menstrual cycles. Therefore, both too much and too little fat are associated with the interruption of their normal reproductive health [78]. Obese women tend to have higher levels of estrogen and prostaglandin and this could be the probable mechanism of dysmenorrhea [64,79]. Additionally, we found that overweight/obese subjects are more likely to suffer from IMC which is comparable to the findings from previous research [24,25,62,80].

In this study, physical activity was a potential risk factor for PMS and IMC which is in line with the results of earlier studies [22,62,81]. Aerobic physical exercise increases hemoglobin, hematocrit, red cell, and platelet count while the levels of prolactin, estradiol, and progesterone decline, which improves fatigue, impaired concentration, confusion, and most PMS [82]. However, a higher level of vigorous physical activity may reduce the risk of IMC but frequent heavy lifting work may increase the chance of IMC [25].

The respondents with a history of COVID-19 infection were at higher risk of having dysmenorrhea and PMS. However, currently, these results cannot be corroborated with other studies. The resulting association of family history with dysmenorrhea and PMS coincides with other findings [17,18,20]. The possible reason could be related to the consequences of genetics and lifestyle followed within the family [5,83].

## Study strengths and limitations

The data quality in this research is quite good as to collect the data, we recruited two female interviewers who are MSc students at the Department of Community Health and Hygiene at PSTU that minimize the bias regard to understand different technical terms. Furthermore, we attempted to investigate the association of major three menstrual disorders viz., dysmenorrhea, PMS, and IMC with the potential risk factors as previously demonstrated in the peer-reviewed literature. Moreover, our targeted respondents were university students to ensure more reliable and valid data as the educated subject can easily understand the questions and provide answers correctly, which minimizes the bias of data collection [84]. Despite these intriguing domains, our study has possessed some limitations. For instance, our data are cross-sectional which may confine our understanding of possible causal intimations. The use of a self-reporting structured questionnaire might introduce response bias, which could result in a discrepancy between reported behaviors and actual practices. To combat this bias, we conducted a pre-test among 50 participants to refine the questionnaire and provided extensive training to ensure neutrality among surveyors. We prioritized participant anonymity, and confidentiality, and obtained informed consent while implementing quality control measures. Further, we accounted for various confounding factors, but we did not consider other potential influences like psychological stress, sleep patterns, and environmental conditions, which might have affected the eumenorrhea. Additionally, our research was confined to female students aged 18 to 26 years at three public universities due to resource constraints. Thus, the findings may not be generalizable to other age groups or the broader female population in Bangladesh.

### Conclusion

Among the surveyed students, dysmenorrhea emerged as the most prevalent menstrual disorder, followed by PMS and IMC. Food craving was identified as a common risk factor while higher intake of vitamin A from plant-based sources was associated with reduced risk of dysmenorrhea and IMC. Additionally, a U-shaped association was observed between BMI and dysmenorrhea; however, overweight/obese students were more likely to experience IMC. This study also found that sedentary physical activity increased the risk of developing PMS and IMC. These findings highlighted the potential for interventions to reduce menstrual disorders among young women attending university, such as promoting healthy eating habits, avoidance of high-fat and sweet foods, and encouraging participation in regular physical activity and mental health services.

## Supporting information

S1 File English-language questionnaire. (DOCX)

S2 File Calculation of dietary diversity score and percentages of respondents who consumed vitamin A-rich plant and animal food sources. (DOCX)

S3 File The research data collected from female university students in Bangladesh. (XLS)

S1 Table The verbal multidimensional scoring system for the measurement of severity of dysmenorrhea. (DOCX)

S2 Table Bivariate analysis for associated risk factors of Dysmenorrhea pain in ordered logistic regression reporting odds ratios. (DOCX)

S3 Table Bivariate analysis for associated risk factors of PMS in logistic regression reporting odds ratios. (DOCX)

S4 Table Bivariate analysis for associated risk factors of IMC in logistic regression reported odds ratio. (DOCX)
