# Supplementary material for: Food craving, vitamin A, and menstrual disorders: A comprehensive study on university female students
Source: PLoS One. 2024 Sep 25;19(9):e0310995. doi: 10.1371/journal.pone.0310995 (PMC11423980; doi:10.1371/journal.pone.0310995)
Supplement: S1 File — (DOCX) [file pone.0310995.s001.docx]

**Supplemental File 1. Questionnaire**

Research Title: Food craving, vitamin A, and menstrual disorders: a comprehensive study on university female students

Objective of the Study: to assess the prevalence of menstrual disorders among female university students in Bangladesh and identify associated risk factors, especially food cravings and vitamin A consumption.

*(The interviewer will introduce herself and take consent of the interviewee before commencing the session)*

**Introductory statement:**

My name is………………, I am working for Mr. Liton Chndra Sen, professor, faculty of Nutrition and Food Science, Patuakhali Science and Technology University. I am assigned as interviewer for the above mentioned PhD project under the department of Food Engineering and Tea Technology, Shahajalal University of Science and Technology and my assignment is to interview teenage female students in order to determine the nature and prevalence of menstrual disorders among university female students at southern coastal belt of Bangladesh. Therefore I am requesting your permission to conduct the interview schedule and it takes usually 15-20 minutes. We are giving the explicit guarantee that you will not identified by your name or functions in any report using information from this interview and your confidentiality as a participant will remain strictly secure. You don't have to be in the survey, but we hope you will agree to answer the questions since your views are important. If I ask you any question you don't want to answer, just let me know and I will go on to the next question or you can stop the interview at any time I am also begging your consent to allow me to take written notes during the interview.

**What is involved in the study?**

You have been selected as a respondent in this survey. I would like to ask you some questions about your menstrual pattern.

**What will you have to do if you agree to participate?**

Since you have been selected as a respondent in this study, I shall be thankful if you provide your valuable response on certain issues. If some questions cause you embarrassment or make you feel uncomfortable, you can refuse to answer them.

**What are the risks and benefits of this study?**

By providing information you will not have any risk whatsoever, rather this will help Mr. Sen to complete his PhD research as well as assist the directorate of health and policy planners to evaluate, strengthen and refocus national effort to improve teenage reproductive health.

**Confidentiality:**

Whatever information you provide will be kept strictly confidential. It will be used for research purposes and will be seen only by staff and researchers at the organizations mentioned.

**Is there any compensation for participating in the study?**

Your participation as an interviewee in this project is voluntary and promises no financial benefit. There is no explicit or implicit coercion whatsoever to participate.

**Right to refuse or withdraw:**

We would like to make assure that the current research project has been reviewed and approved by Ethical Approval Committee at Shahajalal University of Science and Technology. As the participation is voluntary, you can choose not to answer any individual question or all of the questions. However, if you feel uncomfortable in any way during the interview session, you have the right to withdraw from the interview.

**Who do I contact if I have a question or problem?**

If you wish to know more about your rights as a participant in this study you may write Mr. Liton Chadra sen, Professor, Department of Community Health and Hygiene, Patuakhali Science and Technology University, Dumki, patuakhali-8602, email- [liton.sen@pstu.ac.be](mailto:liton.sen@pstu.ac.be), mobile no. +8801717504808. If you have further questions regarding the nature of this study you may also contact Professor Dr. G.M. Rabiul Islam, Chairman Department of Food Engineering and Tea Technology, Shahajalal University of Science and Technology, Sylhet, email- [rabiat14@yahoo.com](mailto:rabiat14@yahoo.com), phone: 880 821 713 850 extn.242 (office); Cell: +88 01787323944.

**At this time, do you want to ask me anything about the interview?**

May we begin the interview now? If yes, please read carefully and sign the consent paper and make a copy of it.

**Consent to publication:**

This is to state that I give my full permission for the publication, reproduction, broadcast and other use of identifiable details, which can include case history and/or details within the text (schedule A, B, C, and D) to be published in any Journal and Article. I confirm that I have seen and been given the opportunity to read the methodology to be published by any journal.

I understand that the published article may be available in both print and on the internet, and will be available to a broader audience through marketing channels and other third parties. Therefore, anyone can read material published in the Journal. I understand that readers may include not only public health professionals and scholarly researchers but also journalists and general members of the public.

I also understand that the information will be published without my personal details and every attempt will be made to ensure anonymity.

I declare, in consequence of granting this permission, that I have no claim on ground of breach of confidence or any other ground in any legal system against the authors and its agents, publishers, successors and assigns in respect of such use of the collected information.

Participant’s Name: ……………………………Signature (or thumb print): …………….. Date: ………

Name of person obtaining consent: ………………………….. Signature: ……………….. Date: ………..

(Must be study investigator or individual who has been designated to obtain consent)

*To be counter-signed and dated electronically for telephone interviews or in the presence of the participant for face to face interviews*

**Copies**: *Once this has been signed by all parties the participant should receive a copy of the signed and dated participant consent form, and the information sheet. A copy of the signed and dated consent form should be placed in the main project file which must be kept in a secure location.*

**Table A: personal detail of the participant**

| Name of the participant |  |
| --- | --- |
| Name of the University |  |
| Home District |  |
| Mobile number (if any) |  |

**Table B: Socio-demographic information**

| **Residence** | **Age** | **Age at Menarche** | **Marital Status** | **Contraceptive use** | |
| --- | --- | --- | --- | --- | --- |
| **1** | **2** | **3** | **4** | **5** | |
| Usually where do you live?   1. With family 2. At student dormitory | How old are you?  (Years) | At what age did your menstruation start for first time?  (Years) | What is your current marital status?   1. Currently married 2. Divorced/   separated   1. Widowed 2. Never married | | If answer is currently married according to column 5 then-  What type of contraceptive method do you use?  (Write the method name) |
|  |  |  |  | |  |

| **Parent’s educational status** | **Parent’s job status** | **Body Mass Index**  **(BMI)** | | | | **Physical activity** |
| --- | --- | --- | --- | --- | --- | --- |
| 6 | 7 | 8 | | | | 9 |
| What is your parent’s educational status?  1. No education  2. Primary incomplete  3. Primary complete  4. Secondary incomplete  5. Secondary complete  6. higher  (Write down 2 digits, 1^st^ for father and 2^nd^ for mother education) | Do your parent currently do?  1.Government job  2. NGO  3. Entrepreneur  4. Retired person  5. Farmer  6. Fisherman  7. Homemaker  Other: (specify)  (Write down 2 digits, 1^st^ for father and 2^nd^ for mother education) | Calculate the BMI by measuring the height (cm) and weight (kg)  (Use first cell for height and second cell for weight) | | | | What type of Physical activity do you perform?   1. sedentary (typical   activities of daily  living with little or  no exercise)   1. Active (Person running one hour daily) 2. Athlete (running, fast cycling, jumping, climbing, swimming, sports etc.) |
|  |  |  | inch |  | kg |  |

**Table C: Menstrual Characteristics**

| **Sl**  **No.** | **Questions and filters** | **Coding categories** | **Code number/answer** |
| --- | --- | --- | --- |
| 1. | Menstrual Cycle Length (Days)  How many days of intervals normally you have between two consecutive menstrual periods? |  |  |
| 2. | Duration of Menstrual flow (Days)  For how many days the menstrual flow last? |  |  |
| 3. | Type of sanitary materials used  What type of sanitary materials do you use during your period? | 1. sanitary pad 2. tampon 3. local materials (soft cloth, bandage cloth, tissue paper etc.) |  |
|  | Amount of menstrual flow or blood loss per period  How many vulval pads or sanitary towels did you change per day during menstruation? |  |  |
| 4. | Assessment of Dysmenorrhea  (Whether the menstrual period is painful, affect daily activity and requires analgesics) | **Grade 0:** not painful and daily activity is unaffected  **Grade1:** painful but seldom inhibits normal activity; analgesics are seldom required; mild pain  **Grade 2:** Daily activity is affected; analgesics required and give sufficient relief so that absence from school is unusual; moderate pain  **Grade 3:** Activity clearly inhibited; poor effect of analgesics; vegetative symptoms (headache, fatigue, vomiting, and diarrhea); severe pain |  |
| 5. | Pre-menstrual Syndrome (PMS): Do you have experienced one or more of the following symptoms around 7-10 days prior to start of your period with cessation of these symptoms during the period?  depression, rapid mood changes, anxiety, irritability, change in appetite, painful or tender breasts, and swelling or bloating of the abdomen | 1. Absent 2. Present |  |
| 7. | With whom did you consult? |  |  |
| 8. | COVID-19 Infection  Did you have any symptoms of COVID-19 | 1. No 2. Yes   (If yes, follow serial no. 11) |  |
| 9. | Menstrual disorder history  Do you have any familial history of menstrual disorders | 1. No 2. Yes |  |

**Table D: Dietary Habit**

1. ***Food craving***

| 1. | Food craving (high fat and sweet foods)  Dou you like to consume high fat and sweet foods? | 1. No 2. 1 |  |
| --- | --- | --- | --- |

1. ***Measuring dietary diversity***

Did you consume any food item from the following groups during last 24 hours?

| Sl  No. | Food Groups | Food items | Coding category  0= No  1=Yes |
| --- | --- | --- | --- |
| 1. | Cereals | rice, corn/maize, wheat, sorghum, millet or any other grains or foods made from these (e.g. bread, noodles, porridge or other grain products- parata, cha-pati, ruti etc. ) |  |
| 2. | White roots and tubers | white potatoes, white yam, mati alu or other foods made from roots |  |
| 3. | Vitamin A rich vegetables and tubers | pumpkin, carrot, squash, or sweet potato that are orange inside + other locally available vitamin A rich vegetables (e.g. red sweet pepper) |  |
| 4. | Dark green leafy vegetables | dark green leafy vegetables, including wild forms + locally available vitamin A rich leaves such as amaranth, spinach etc. |  |
| 5. | Other vegetables | other vegetables (e.g. tomato, onion, eggplant) + other locally available vegetables |  |
| 6. | Vitamin A rich fruits | ripe mango, ripe papaya, palm , and 100% fruit juice made from these + other locally available vitamin A rich fruits (orange inside) |  |
| 7. | Other fruits | other fruits, including wild fruits and 100% fruit juice made from these |  |
| 8. | Organ meat | liver, kidney, heart or other organ meats or blood-based foods |  |
| 9. | Flesh meats | beef, lamb, mutton, chicken, duck, other birds and insects |  |
| 10. | Eggs | eggs from chicken, duck quail or any other egg |  |
| 11. | Fish and seafood | fresh or dried fish or shellfish |  |
| 12. | Legumes, nuts and seeds | dried beans, dried peas, lentils, nuts, seeds or foods made from these (eg. peanut butter) |  |
| 13. | Milk and milk products | milk, cheese, yogurt or other milk products |  |
