# Supplementary material for: Food craving, vitamin A, and menstrual disorders: A comprehensive study on university female students
Source: PLoS One. 2024 Sep 25;19(9):e0310995. doi: 10.1371/journal.pone.0310995 (PMC11423980; doi:10.1371/journal.pone.0310995)
Supplement: S2 File — (DOCX) [file pone.0310995.s002.docx]

**Supplemental File 2. Calculation of dietary diversity score and percentages of respondents who consumed vitamin A-rich plant and animal food sources:**

The dietary data was collected based 24 hours recall system and Women’s Dietary Diversity Score (WDDS) had been calculated according to guideline developed by Food and Agricultural Organization (FAO) in 2011[1].

Table A: the respondents were asked to recall their consumption of any food item consumed from the following groups during last 24 hours? (N=391).

| Serial no. of groups | Food Groups | Food items | If No= 0  and  If yes=1 |
| --- | --- | --- | --- |
| 1. | Cereals | rice, corn/maize, wheat, sorghum, millet or any other grains or foods made from these (e.g. bread, noodles, porridge or other grain products- parata, cha-pati, ruti etc. ) |  |
| 2. | White roots and tubers | white potatoes, white yam, mati alu or other foods made from roots |  |
| 3. | Vitamin A-rich vegetables and tubers | pumpkin, carrot, squash, or sweet potato that are orange inside + other locally available vitamin A rich vegetables (e.g. red sweet pepper) |  |
| 4. | Dark green leafy vegetables | dark green leafy vegetables, including wild forms + locally available vitamin A rich leaves such as amaranth, spinach etc. |  |
| 5. | Other vegetables | other vegetables (e.g. tomato, onion, eggplant) + other locally available vegetables |  |
| 6. | Vitamin A-rich fruits | ripe mango, ripe papaya, palm , and 100% fruit juice made from these + other locally available vitamin A rich fruits (orange inside) |  |
| 7. | Other fruits | other fruits, including wild fruits and 100% fruit juice made from these |  |
| 8. | Organ meat | liver, kidney, heart or other organ meats or blood-based foods |  |
| 9. | Flesh meats | beef, lamb, mutton, chicken, duck, other birds and insects |  |
| 10. | Eggs | eggs from chicken, duck quail or any other egg |  |
| 11. | Fish and seafood | fresh or dried fish or shellfish |  |
| 12. | Legumes, nuts and seeds | dried beans, dried peas, lentils, nuts, seeds or foods made from these (eg. peanut butter) |  |
| 13. | Milk and milk products | milk, cheese, yogurt or other milk products |  |

All the food items (from Table A) consumed by the respondents were merge into nine common food groups for the calculation of women dietary diversity score (WDDS) as follows:

1. Starchy staples (1 and 2)* : cereals; white roots and tubers
2. Other vitamin A-rich fruits and vegetables (3 and 6)*: vitamin A-rich vegetables and tubers; as well as vitamin A-rich fruits
3. Dark green leafy vegetables (4)*
4. Other fruits and vegetables (5 and 7)*: Other vegetables and other fruits
5. Meat and fish (9 and 11)*: flesh meats; fishes and seafood
6. Organ meat (8)*
7. Eggs (10)*
8. legumes, nuts and seeds (12)*
9. Milk and milk products (13)*

**indicating the number of food groups from the above* ***Table A*** *which were merge into a single group of foods for analysis.*

The diversity score range was 0-9 whereas the value for the consumption of each food group was either 0 “No” or 1 “Yes”. Different level of dietary diversity score were classified as i) lowest dietary diversity (≤ 3 food groups), ii) medium dietary diversity (4-5 food groups) and iii) high dietary diversity (≥ 6 food groups).

***Calculation of Vitamin A-rich food sources***

Individuals who consumed at least one plant-based food group high in vitamin A, such as dark green leafy vegetables or other vitamin A-rich fruits and vegetables, received a score of 1. Those who did not consume any of these vitamin A-rich food groups were given a score of 0. Additionally, subjects who consumed at least one vitamin A-rich animal food group, such as organ meat, eggs, or dairy products, were scored as 1. Those who did not consume any of these animal-based foods were given scored as 0 [1].

Table B: Calculation of Vitamin A-rich food sources

| **Food sources of vitamin A** | **Number of Food groups (see above)** | **Food groups** |
| --- | --- | --- |
| Plant based | ii | dark green leafy vegetables |
|  | iii | Other vitamin A-rich fruits and vegetables |
| Animal based | vi | Organ meat |
|  | vii | Eggs |
|  | ix | Milk and milk products |

The percentage of individuals who consumed vitamin A rich plant food sources during the last 24 hours was calculated using the following formula:

$$\frac{Number of individuals who consumed dark green leafy vegetables OR other vitamin A rich fruits and vegetables}{Total number of respondents}x 100$$

The percentage of respondents who consumed vitamin A rich animal food sources during the last 24 hours was calculated as follows:

$$\frac{Number of individuals who consumed organ meat OR eggs OR milk and milk products}{Total number of respondents}x 100$$

Reference:

1. Kennedy G, Ballard T, Dop MC. Guidelines for measuring household and individual dietary diversity: Food and Agriculture Organization of the United Nations; 2011.
