# Supplementary material for: Food craving, vitamin A, and menstrual disorders: A comprehensive study on university female students
Source: PLoS One. 2024 Sep 25;19(9):e0310995. doi: 10.1371/journal.pone.0310995 (PMC11423980; doi:10.1371/journal.pone.0310995)
Supplement: S1 Table — (DOCX) [file pone.0310995.s004.docx]

**Supplemental Table 1. The verbal multidimensional scoring system for the measurement of severity of dysmenorrhea (1)**

| **Grade** | **Working ability** | | **Systemic symptoms** | **Analgesics** |
| --- | --- | --- | --- | --- |
| **Grade 0:** Menstruation is not painful and daily activity is unaffected. No pain | Unaffected | | None | Not required |
| **Grade 1:** Menstruation is painful but seldom inhibits the women activity. Analgesics are seldom required. Mild pain | | Rarely affected | None | Rarely required |
| **Grade 2:** Daily activity is affected; analgesics required and give sufficient relief so that absence from school is unusual. Moderate pain | Moderately affected | | Few | Required |
| **Grade 3:** Activity clearly inhibited; poor effect of analgesics; vegetative symptoms (headache, fatigue, vomiting, and diarrhea). Severe pain | Clearly  inhibited | | Apparent | Poor effect |

**Reference:**

1. Andersch B, Milsom I. An epidemiologic study of young women with dysmenorrhea. American journal of obstetrics and gynecology. 1982;144(6):655-60.
