# Supplementary material for: Food craving, vitamin A, and menstrual disorders: A comprehensive study on university female students
Source: PLoS One. 2024 Sep 25;19(9):e0310995. doi: 10.1371/journal.pone.0310995 (PMC11423980; doi:10.1371/journal.pone.0310995)
Supplement: S2 Table — (DOCX) [file pone.0310995.s005.docx]

**Supplemental Table 2. Bivariate analysis for associated risk factors of Dysmenorrhea pain in ordered logistic regression reporting odds ratios (N=391)**

| **Predictable variables** | **COR (95% CI)** | **P Value** |
| --- | --- | --- |
| ***Food craving (High fat and sweet food)*** |  |  |
| No (ref.) | - |  |
| Yes | 3.8 (2.4 – 6.08) | <0.001*** |
| ***Consume Vitamin A rich plant food sources*** |  |  |
| No (ref.) | - |  |
| Yes | 0.4 (0.3 – 0.6) | <0.001*** |
| ***Mother's educational status*** |  |  |
| Secondary/Higher (ref.) | - |  |
| Below secondary | 3.6 (2.4 – 5.4) | <0.001*** |
| ***Place of residence*** |  |  |
| With family (ref.) | - |  |
| At student dormitory | 4.3 (2.3 – 8.1) | <0.001*** |
| ***Age at menarche*** |  |  |
| > 12 years (ref.) | - |  |
| ≤ 12 years | 2.0 (1.3 – 2.9) | 0.001** |
| ***BMI (kg/m^2^)*** |  |  |
| Normal weight ( 18.5-22.9) (ref.) | - |  |
| Underweight (<18.5) | 3.09 (1.8 – 5.3) | <0.001*** |
| Overweight/Obese (>22.9) | 2.9 (1.9 – 4.5) | <0.001*** |
| ***Infected by COVID19 disease*** |  |  |
| No (ref.) | - |  |
| Yes | 2.0 (1.3 – 3.06) | 0.003** |
| ***Family history of menstrual disorders*** |  |  |
| No (ref.) | - |  |
| Yes | 3.0 (1.9 – 4.7 ) | <0.001*** |

*COR= Crude Odd Ratio, * indicated the level of significance i.e. * p<0.05, ** p<0.01and ***p<0.001*
