# Supplementary material for: Food craving, vitamin A, and menstrual disorders: A comprehensive study on university female students
Source: PLoS One. 2024 Sep 25;19(9):e0310995. doi: 10.1371/journal.pone.0310995 (PMC11423980; doi:10.1371/journal.pone.0310995)
Supplement: S3 Table — (DOCX) [file pone.0310995.s006.docx]

**Supplemental Table 3. Bivariate analysis for associated risk factors of PMS in logistic regression reporting odds ratios (N=391)**

| **Predictable variables** | **COR (95% CI)** | **P Value** |
| --- | --- | --- |
| ***Food craving (High fat and sweet food)*** |  |  |
| No (ref.) | - |  |
| Yes | 4.6 (2.8 – 7.6) | <0.001*** |
| ***Age at menarche*** |  |  |
| > 12 years (ref.) | - |  |
| ≤ 12 years | 1.7 (1.1 – 2.7) | 0.016* |
| ***Physical activity level*** |  |  |
| Active and Athlete (ref.) | - |  |
| Sedentary | 2.7 (1.5 – 4.9) | 0.002** |
| ***Infected by COVID19 disease*** |  |  |
| No (ref.) | - |  |
| Yes | 2.2 (1.4 – 3.5) | 0.002** |
| ***Family history of menstrual disorders*** |  |  |
| No (ref.) | - |  |
| Yes | 5.5 (3.3 – 9.2 ) | 0.000*** |

*COR= Crude Odd Ratio, * indicated the level of significance i.e. * p<0.05, ** p<0.01and ***p<0.001*
