# Supplementary material for: Food craving, vitamin A, and menstrual disorders: A comprehensive study on university female students
Source: PLoS One. 2024 Sep 25;19(9):e0310995. doi: 10.1371/journal.pone.0310995 (PMC11423980; doi:10.1371/journal.pone.0310995)
Supplement: S4 Table — (DOCX) [file pone.0310995.s007.docx]

**Supplemental Table 4. Bivariate analysis for associated risk factors of IMC in logistic regression reported odds ratio (N = 391)**

| **Predictable variables** | **COR (95% CI)** | **P Value** |
| --- | --- | --- |
| ***Food craving (High fat and sweet food)*** |  |  |
| No (ref.) | - |  |
| Yes | 4.3 (2.6 – 7.2) | 0.000*** |
| ***Consume Vitamin A rich plant food sources*** |  |  |
| No (ref.) | - |  |
| Yes | 0.2 (0.2 – 0.4) | 0.000*** |
| ***Consume Vitamin A rich animal food sources*** |  |  |
| No (ref.) | - |  |
| Yes | 2.3 (1.3 – 3.9) | 0.004** |
| ***Marital status*** |  |  |
| Ever married | - |  |
| Never Married | 2.5 (0.9 – 7.2) | 0.098 |
| ***BMI(kg/m^2^)*** |  |  |
| Normal weight ( 18.5-22.9) (ref.) | - |  |
| Underweight (<18.5) | 2.2 (1.1 – 4.3 ) | 0.023* |
| Overweight/Obese (>22.9) | 4.4 (2.5 – 7.4) | 0.000*** |
| ***Physical activity level*** |  |  |
| Active and Athlete (ref.) | - |  |
| Sedentary | 4.2 (1.9 – 9.5) | 0.001** |

*COR= Crude Odd Ratio, * indicated the level of significance i.e. * p<0.05, ** p<0.01and ***p<0.001*
